# How Does Leadership in Safety Management Affect Employees’ Safety Performance? A Case Study from Mining Enterprises in China

**DOI:** 10.3390/ijerph19106187

**Published:** 2022-05-19

**Authors:** Shu Zhang, Xinyu Hua, Ganghai Huang, Xiuzhi Shi

**Affiliations:** 1 School of Resources and Safety Engineering, Central South University, Changsha 410083, China; zhangshu@csu.edu.cn (S.Z.); 205512131@csu.edu.cn (X.H.); baopo@csu.edu.cn (X.S.); 2 School of Civil Engineering, Central South University, Changsha 410083, China

**Keywords:** safety leadership, safety performance, safety compliance, safety participation

## Abstract

Leadership is a necessary element for ensuring workplace safety. Rather little is known about the role of leadership safety behaviours (LSBs) in the mining industry. Using regression analysis and structural equation modelling analysis, this study examined the cause-and-effect relationships between leadership safety behaviours and safety performance. Data were collected by questionnaires from 305 miners in China. Data were analysed using exploratory factor analysis and confirmatory factor analysis, which identified five main dimensions of LSBs: safety management commitment, safety communication with feedback, safety policy, safety incentives, and safety training; the analysis also identified three main dimensions of safety performance: employee’s safety compliance, safety participation, and safety accidents. The results showed the overall effects of each LSB variable on safety compliance in descending order as: safety training (0.504), safety incentives (0.480), safety communication with feedback (0.377), safety management commitment (0.281), and safety policy (0.110). The overall effects of each LSB variable on safety participation in descending order were: safety training (0.706), safety incentives (0.496), safety management commitment (0.365), and safety policy (0.247). Furthermore, we found that safety management commitment and safety incentives increased employees’ safety behaviours, but this influence was mediated by safety training, safety policy, and safety communication with feedback.

## 1. Introduction

According to the State Administration of Work Safety in China [1], 5930 accidents and 18,567 deaths occurred in the national mines from 2003 to 2021, second only to the transport industry. These startling facts tell us that the mine safety situation is still grim in China. In 2019, in Inner Mongolia province, accidents while transporting workers down into mines caused 20 deaths and 30 injuries. In 2021, in Shanxi province, a mine flood caused 13 deaths. The same year, in Shandong province, 10 people died, 11 were injured, and 1 was missing in a blast in a return air shaft. Moreover, in addition to China, serious mining accidents have occurred in other countries. In 2021 in Kuzbas, Russia, mining fires occurred that resulted in 52 deaths. In 2022 in Poland, mine collapses resulted in 10 deaths, and a few days later, methane exploded in a coal mine and led to 8 deaths and 7 missing. In addition to the victims’ psychological and physical harm, heavy casualties bring families tremendous suffering. From this situation arises two questions: What are the main causes of accidents, and how can safety be improved? 

The causes of accidents are complex and closely related to safety management practices [2]. With the growing understanding of the role of safety behaviours in the accident investigation process, people are gradually paying more attention to management deficiencies and leadership behaviours (LSBs). The current research on safety behaviour has made two breakthroughs. On the one hand, it has broadened the range of behaviours beyond employees’ working safety behaviours to focus on leaders’ management behaviours. For example, Conchie et al. explored factors influencing supervisors’ safety leadership in construction [3]. In a survey of about 10,000 workers employed on offshore platforms operating on the Norwegian, Dahl and Olsen indicated that safety leadership affected workers’ safety performance through the intervening variable of work climate [4]. Li found that effective mining safety management can prevent accidents [5], and his team analysed the relationship between safety leadership and leadership style [6]. A study focused on Portuguese companies found that safety management behaviours, such as OHS training, largely affected workers’ behaviours [7]. On the other hand, the research investigated in depth factors affecting safety behaviour, which included not only the individual’s physiological and psychological factors [8] but also organizational factors [9], environmental factors [10,11], and leadership factors [3,4,5]. This paper mainly studies the LSBs first, then concerns about the consequences of LSBs that influence miners’ safety performance.

At present, a large number of studies have shown that LSBs have major impacts on employees’ safety behaviour. Generally, leadership safety behaviours (LSBs) were defined as behaviours exhibited by managers when focusing on safety performance [12]. Barling et al. [13] found that supervisors’ transformational leadership is positively related to employees’ safety behaviours in the hospitality sector, which showed that employees’ perceptions of transformational leadership can determine their self-reported safety behaviours. S. Larsson et al. [14] investigated how individual psychological atmosphere affected safety behaviour, again finding that management practices can change employees’ safety behaviours. Lu and Yang [15] studied the relationships between safety leadership and employees’ self-reported safety behaviours in the container shipping context, and the results suggested that safety incentives, safety policy, and safety concerns positively affected employees’ safety participation. Sampling 548 railroad workers, Kath [16] et al. concluded that the communication between workers and superiors has a close relationship with unsafe behaviours. A previous study also found that the core leadership behaviours that positively influenced safety included continuous planning and coordination, role modelling, monitoring work, and proactively correcting deviations [17]. At present, these studies have been conducted in various industries, such as air transportation [18], medical industry [19], mining industry [20], construction [21], electricity industry [22], chemical industry [23], and manufacturing [24].

In a variety of fields, studies on the growing concerns about safety leadership have demonstrated that safety leadership increases organizations’ safety performance. Developing and maintaining safety leadership is critical for reducing accidents and promoting safety, and the main purpose of this paper is to explore the relationships between LSBs and employees’ safety behaviours in mining enterprises.

### 1.1. Leadership Safety Behaviours (LSBs)

Many scholars studied aspects of LSBs such as safety management commitment, safety communication with feedback, safety policy, safety incentives, and safety training. Safety management commitment reflects leaders’ attitudes toward safety. It plays an important role in organizations’ safety programmes [25]. We can understand safety commitment by leaders’ safety awareness. Their commitment is reflected in safety rules, regulations, and policies; safety responsibilities; emergency responses; and human, financial, and material resources [26]. Conducting a survey in India, Vinodkumar found that workers at companies certified by OHSAS 18001 and ISO 9001 show more commitment to safety behaviours [27]. Plausible safety commitment from leaders may influence workers’ psychological capital, in turn improving their safety performance [28]. Good communication and feedback will help employees gain experience and promote safety awareness effectively in the event of accidents. Regular communication between managers and employees can also improve the safety atmosphere in workplaces. Some studies showed that safety communication with feedback significantly affected organizational safety [16,28]. Konstantin et al. also indicated that safety communication greatly affected workers’ safety behaviours [29]. Following a literature review, Cohen concluded that abundant safety communication raises workers’ safety awareness [30]. Carrillo and Simon [31] and Barling et al. [13] considered safety communication with feedback one dimension of LSBs. Other researchers, such as Hofmann and Morgeson [32], Mearns et al. [33], Probst [34], Zuo [35], and Oswald and Lingard [36], also confirmed that safety communication was negatively related to occupational accidents. Safety policy consists of safety regulations and procedures and represents the safety standards of enterprises. Reasonable safety policies help employees clearly understand safety requirements and thus improve employees’ safety behaviours [15,27,37,38]. Safety incentives also reflect one dimension of LSBs. Appropriate safety incentives can motivate and strengthen employees’ safety behaviours [15,39,40]. As such, establishing a fair evaluation and reward system can effectively avoid unsafe behaviours. For enterprise managers and ordinary employees, safety management effects are inseparable from those of safety education and training. Safety training can not only improve employees’ skills in risk identification and crisis management but also improve their safety knowledge and awareness. Improper safety training negatively affects employees’ safety behaviours [41,42].

### 1.2. Enterprise Safety Performance (ESP)

The traditional assessment of safety performance has been mainly based on accidents, near-misses, injuries, diseases, and other objective results. Currently, safety performance also includes safety behaviours and accident rates. Neal et al. [43] considered safety compliance and safety participation components of safety performance. Through exploring the influencing factors of safety performance, Abbas [44] found that the relationships among managers, harmonious environments, and work flexibility significantly influenced safety performance. From organizational levels, Claudio et al. [45] identified two elements and two important influencing factors for safety performance: safety participation, safety norms, safety habits, and safety compliance. The lean philosophy developed to improve safety management and safety performance [46], and Cordeiro et al. identified that lean tools—5S, visual management, and OPL—are important for improving safety conditions and promoting a safety culture as part of safety management [47].

### 1.3. Research Hypothesis

To summarize the literature findings, a considerable number of domestic and foreign scholars have studied LSBs and obtained many valuable research results. Prior studies generally agreed that LSBs included safety management commitment, safety communication with feedback, safety policy, safety incentives, and other dimensions; most of the studies used surveys. At the same time, many scholars studied how LSBs affect employees’ safety behaviours. Whether a safety culture or climate has successfully been established has a vital link with leaders’ attitudes, behaviours, and decision-making. Leaders’ words greatly influence employees’ views and ideas, and we argue that LSBs include some dimensions of safety culture or climate.

Although management and production are different in the mining industry than in other industries, there are some similarities such as the high risks of construction, managers’ high safety responsibilities in marine transportation, and the need for specific safety measures such as in machinery and chemical industries. Based on the existing research, we aimed to study five dimensions of LSBs: safety management commitment (SMC), safety communication with feedback (SCF), safety policy (SP), safety incentives (SI), and safety training (ST), and three dimensions of enterprise safety performance, safety compliance, safety participation, and safety accidents.

Based on the literature, we proposed the following hypotheses:

**Hypothesis 1** **(H1).** *Among LSBs, safety training (1), safety management commitment (2), safety incentives (3), safety policy (4), and safety communication with feedback (5) will affect safety compliance directly, significantly, and positively.*

**Hypothesis 2** **(H2).** *Among LSBs, safety training (1), safety management commitment (2), safety incentives (3), safety policy (4), and safety communication with feedback (5) will affect safety participation directly, significantly, and positively.*

**Hypothesis 3** **(H3).** *Among LSBs, safety training (1), safety management commitment (2), safety incentives (3), safety policy (4), and safety communication with feedback (5) will affect safety accidents directly, significantly, and positively.*

## 2. Materials and Methods

### 2.1. Sample

For this study, we conducted a questionnaire survey in lead-zinc mines in China. The survey was sent to 450 employees in middle, primary, and workshop. A total of 450 questionnaires were returned, for a response rate of 100%. A total of 145 invalid questionnaires were collected, and 305 valid questionnaires were obtained, an effective rate of 68%. Thus, the number of samples satisfied statistical requirements.

### 2.2. Measures

#### 2.2.1. Independent Variables

The employees’ perceptions about the five dimensions of LSBs were measured with a questionnaire based on a review of related literature and theories [31,39,48,49]. The questionnaire contained questions covering safety management commitment (9 items), safety communication with feedback (5 items), and safety training (6 items) designed with reference to previous studies [27,28,37,43,50,51,52]. In addition, it contained questions covering safety policy (4 items) and safety incentives (6 items) which referred to Cooper [39], Carrillo and Simon [31], O’Dea and Flin [48], Wu et al. [49], and Lu and Yang [15]. This portion of the questionnaire had a total of 30 items (see Appendix A). In this research, respondents rated items on five-point Likert scales (anchored by 1 = “strongly disagree” and “5 = strongly agree”).

#### 2.2.2. Dependent Variables

ESP was measured on three dimensions: safety compliance, safety participation, and safety accidents. In previous studies, self-reported safety behaviour referred to safety compliance and safety participation. Based on the literature review, we adapted safety compliance (4 items) and safety participation (3 items) from Borman and Motowidlo [53], Neal et al. [43], and so on. We added one item to the dimension of safety compliance: “In order to complete more work to get more piece-rate income or measurement of income, I may ignore safety”. This question acknowledged the actual mining situation in China. In addition, safety incidents were measured with the item “In the past three years, I have been in an accident”, taken from Leung et al. [42]. In total, the safety performance questionnaire consisted of 9 items (see Appendix B). Respondents assessed items using five-point Likert scale.

#### 2.2.3. Control Variables

In addition to LSBs and safety performance, the questionnaire included some control variables. Respondents’ sex, age, education level, work experience, job position, and frequency of safety training were the control variables. Education level and job position reflected the respondents’ understanding of safety leadership and safety behaviours. The frequency of safety training indicated whether respondents had abundant safety knowledge, which would affect their understanding of safety behaviours.

### 2.3. Data Analysis

Firstly, exploratory factor analysis was used to confirm the dimensions of LSBs and safety performance from the questionnaire. Then, confirmatory factor analysis was used to examine the reliability and validity of the questionnaires on LSBs and safety performance. Next, regression analysis was conducted to explore LSBs’ influence on safety performance. Last, SEM analysis was conducted to study LSBs’ influencing mechanism on safety performance.

#### 2.3.1. Questionnaire of LSBs

##### Exploratory Factor Analysis

Based on 50% of the sample, SPSS16.0 was used for exploratory factor analysis. Principal component analysis with varimax rotation was employed to identify the dimensions of LSBs. The results showed that only variables with a factor loading greater than 0.50 were extracted; the retained items’ factor loadings ranged from 0.527 to 0.907. The total explained variance of the common LSB factors amounted to 70.897%, and Cronbach’s α > 0.60, which indicated that the five dimensions of LSBs were persuasive. Through exploratory factor analysis, a total of 13 items were deleted: X4, X5, X6, X7, X11, X12, X14, X15, X18, X21, X23, X24, and X28. Thus, a five-dimensional structure was formulated. Then, factor analysis was conducted to confirm the reliability and stability of the five dimensions of LSBs.

Factor 1 was called safety training and accounted for 59.66% of the total variance. Factor 2 was called safety management commitment and accounted for 18.335% of the total variance. Factor 3 was designated safety incentives and accounted for 12.697% of the total variance. Factor 4 was called safety policy and accounted for 9.307% of the total variance. Factor 5 was designated safety communication with feedback and accounted for 9.119% of the total variance.

##### Confirmatory Factor Analysis

Structural equation modelling (SEM) confirmatory factor analysis was used to study the reliability and validity of the LSB questionnaire [54].

(1) Fit test of the structural model

**Figure 1:**
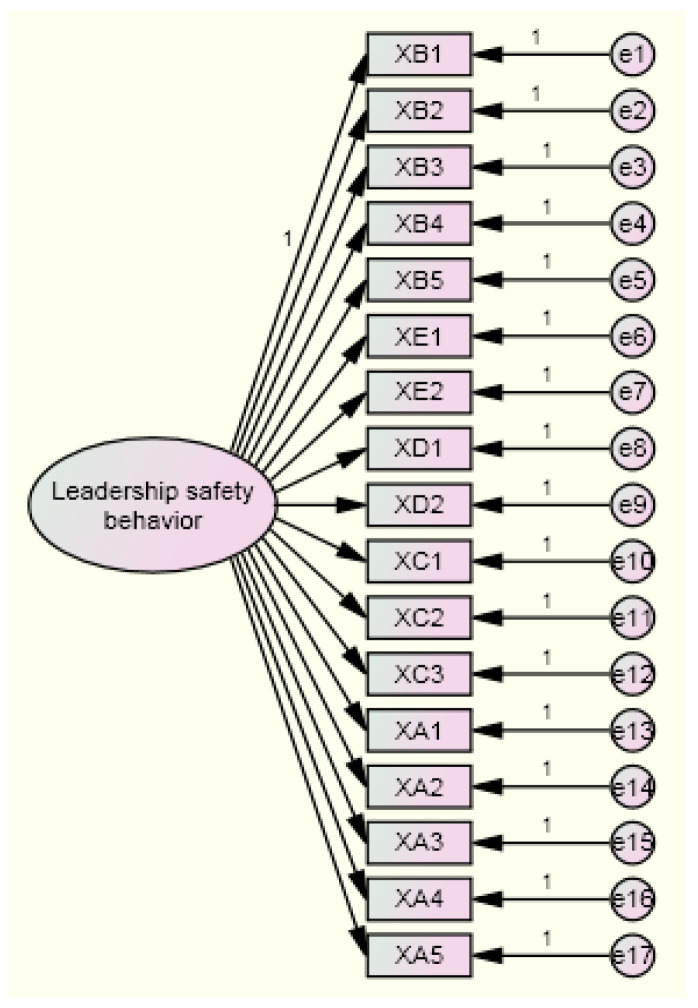
The single-factor structural model.

**Figure 2:**
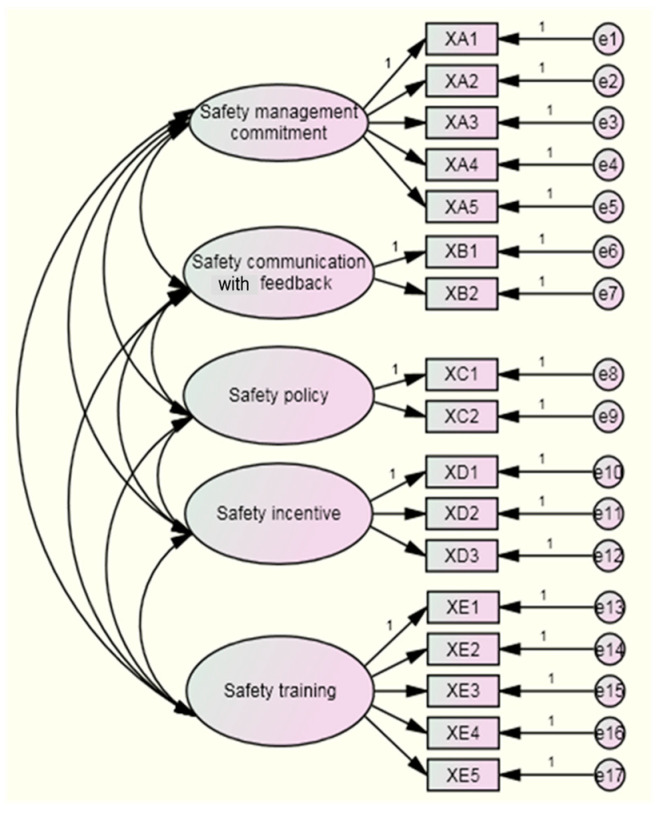
The five-factor structural model.

Following the results of exploratory factor analysis, two models for hypothesis testing were proposed: a single-factor model and a five-factor model (Figure 1 and Figure 2).

The results showed a chi-square degrees of freedom ratio (χ^2^/df = 1.205) of less than 2 and an RMSEA (0.038) of less than 0.08 that indicated that the five-factor structure model fit better. At the same time, although the AGFI of the five-factor model did not meet 0.90, the model also did not have a negative error variable. All factor loadings ranged from 0.50 to 0.95, the standard error was fair, all parameters were significant, and the standardized residuals with absolute value were less than 2.58. Overall, the five-factor model fit well and had good construct validity. Thus, the five-factor model could be accepted.

(2) Reliability

The reliability of the structural model could be assessed by Cronbach’s α and construct reliability. Generally, Cronbach’s α more than 0.60 yields high confidence of a model. Construct reliability more than 0.6 indicates ideal intrinsic quality of the model and that the measurement tool is stable. The results showed that the Cronbach’s α of the latent variables ranged from 0.616 to 0.869 (>0.6), and the construct reliability ranged from 0.656 to 0.873 (>0.6). The measurements of each latent variable had good internal consistency.

(3) Validity

Convergent validity can be confirmed by *t*-values which are all statistically significant on the factor loadings. In the AMOS text output file, the *t*-value is the critical ratio (C.R.). The larger the factor loadings or coefficients, the stronger the observed variables’ representation of their specified latent variables. Each item exceeds the critical ratio at the 0.05 level of significance (*p*). Thus, all observed variables were significantly related to their specified latent variables. Item reliability refers to the *R*^2^ value, which can be used to estimate the reliability of a particular observed variable (item). *R*^2^ > 0.50 provides evidence for acceptable reliability. Although, some items’ *R*^2^ values were less than 0.50, generally speaking, the relationships between the latent variables and the observed variables were reasonable, and this model had good convergent validity.

Discriminant validity can be measured by comparing the goodness of fit before and after the merger of two factors. The results showed the discriminant validity between 10 paired factors. The difference of chi-square value between the restricted model and the unrestricted model reached the 0.05 level of significance. The results provided evidence of good discriminant validity.

In addition, the variance extracted can also measure construct reliability. High variance extracted values occur when the observed variables are truly representative of the latent variables. The results showed that the variance extracted values are: 0.476, 0.511, 0.576, 0.583, and 0.626. Overall, most of the latent variables had a variance extracted value that was higher than the recommended level of 50%. This indicated that the overall goodness-of-fit results supported the proposed model.

Based on the results above, the five-factor LSB model had high reliability and validity and could be used for further research.

#### 2.3.2. Questionnaire of Safety Performance

Using AMOS18.0, confirmatory factor analysis was used to examine the reliability and validity of the safety performance questionnaire. The calculation methods were similar to the foregoing.

The results showed that the chi-square degrees of freedom ratio (χ^2^/df = 1.494) was less than 2 and the RMSEA (0.059) was less than 0.08, indicating that the three-factor model of safety performance fit well. Cronbach’s α of the latent variables were 0.790 and 0.806 (>0.6), and the construct reliability values were 0.765 and 0.806 (>0.5). Hence, the measurements of each latent variable had good internal consistency. For convergent validity, the standardized factor loadings of all items reached the level of significance, which indicated that the questionnaire had high convergent validity.

Based on the results above, the three-factor model of safety performance, with high reliability and validity, could be used for further research.

#### 2.3.3. Regression Analysis

Regression analysis was used to study the relationships between LSBs and safety performance, including simple linear regression analysis and stepwise multiple regression analysis [55].

First, simple linear regression analysis was conducted with the five dimensions of LSBs as independent variables and safety performance as the dependent variable. The results showed that safety training, safety management commitment, safety incentives, safety policy, and safety communication with feedback significantly positively predicted safety performance, accounting for, respectively, 76.5%, 62.5%, 49.6%, 49.9%, and 25.1% of the variance in safety performance (see Table 1). This indicates that the better the safety training, the better the employees’ safety performance, which is the same as safety management commitment, safety incentives, safety policy, and safety communication with feedback.

Then, stepwise multiple regression analysis was conducted with the five dimensions of LSBs as independent variables and safety performance as the dependent variable. Three predictor variables, safety training, safety communication with feedback, and safety policy, significantly predicted safety performance (*R* = 0.765, *R*^2^ = 0.585, *F* = 64.879 ***). That is, a total of three predictor variables effectively explained 58.5% of the variance in safety performance. Safety training had the largest predictive power with 52.3% of the variance (see Table 2). The standardized regression coefficients (Beta) of the three predictor variables were positive, which demonstrated that the variables positively affected safety performance. However, separate regression analysis revealed relationships of safety management commitment with safety performance and safety incentives with safety performance, meaning that these two variables were significant positive predictors of safety performance. In turn, this finding meant that when safety training, safety policy, and safety communication with feedback were added to the relationships between safety management commitment, safety incentives and safety performance, safety management commitment and safety incentives showed significantly lower predictive power for safety performance, which indicated that the first three factors likely played significant mediating effect.

#### 2.3.4. SEM Analysis

Regression analysis indicated the partial mediation of safety training, safety policy, and safety communication with feedback. Therefore, considering safety training, safety policy, and safety communication with feedback as mediator variables, the effects of LSBs on safety performance were further studied by SEM [56]. The following hypotheses were proposed:

**Hypothesis 4** **(H4).** *Safety training (1), safety policy (2), and safety communication with feedback (3) will mediate the relationship between safety management commitment and employee safety compliance.*

**Hypothesis 5** **(H5).** *Safety training (1), safety policy (2), and safety communication with feedback (3) will mediate the relationship between safety management commitment and employee safety participation.*

**Hypothesis 6** **(H6).** *Safety training (1), safety policy (2), and safety communication with feedback (3) will mediate the relationship between safety management commitment and safety accident.*

**Hypothesis 7** **(H7).** *Safety training (1), safety policy (2), and safety communication with feedback (3) will mediate the relationship between safety incentive and employee safety compliance.*

**Hypothesis 8** **(H8).** *Safety training (1), Safety policy (2), and Safety communication with feedback (3) will mediate the relationship between safety incentive and employee safety participation.*

**Hypothesis 9** **(H9).** *Safety training (1), Safety policy (2), and Safety communication with feedback (3) will mediate the relationship between safety incentive and safety accident.*

**Figure 3:**
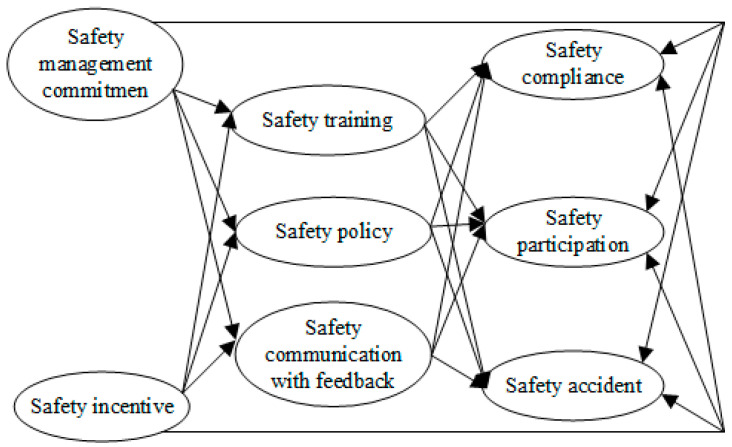
The partial mediation model of leadership behaviours’ effects on safety performance (the first model).

**Figure 4:**
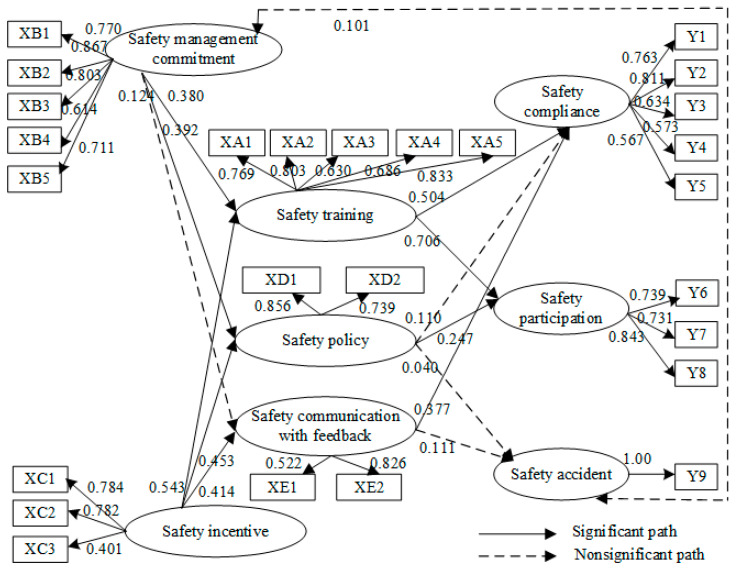
The revised partial mediator model of leadership behaviours’ effect on safety performance (the second model).

To test the hypotheses above, a structural model was established through SEM (Figure 3). There were eight latent variables in the structural model: safety management commitment, safety communication with feedback, safety policy, safety incentives, safety training, safety compliance, safety participation, and safety accidents. The first model fit better (χ^2^/df = 1.102 < 2, RMSEA = 0.027 < 0.08, see Figure 3) and was revised by deleting the negative paths (Figure 4).

In the revised model (χ^2^/df = 1.126 < 2, RMSEA = 0.030 < 0.08), safety management commitment had no significant effect on safety compliance, safety participation, or safety accidents. However, when the three mediators, safety training, safety policy, and safety communication with feedback, were added, safety management commitment and safety incentives significantly influenced safety compliance and safety participation (Figure 4). This result confirmed the mediation hypotheses above. Thus, the fifth model was selected. Next, the effects of the five dimensions of LSBs on the three dimensions of safety performance will be further analysed.

## 3. Results

Based on the results of SEM analysis, we illustrated the effect of LSBs on safety performance through the standardized path coefficients; the direct, indirect, and overall effects; and the mediator effects.

### 3.1. Standardized Path Coefficient

If C.R. > 1.96, the path is significant (see Table 3). The results showed that safety management commitment (SMC) significantly and positively affects safety training (ST, C.R. = 4.314) and safety policy (SP, C.R. = 4.001), which is in line with the hypothesis. That is, leaders’ convincing safety management commitment and emphasis on safety at work improves safety training and safety policy. In addition, safety management commitment does not significantly affect safety communication with feedback (SCF, C.R. = 1.066).

Safety incentives (SI) significantly and positively affect safety training (ST, C.R. = 5.456), safety policy (SP, C.R. = 4.488), and safety communication with feedback (SCF, C.R. = 2.821), consistent with the hypothesis. The finding indicates that proper and compelling safety incentives can improve the effects of safety training and safety policy and improve safety communication.

Safety training (ST) significantly and positively affects safety compliance (C.R. = 4.952) and safety participation (C.R. = 6.575): Regular and effective safety training improves employees’ safety compliance and increases their participation in safety activities.

Safety communication with feedback (SCF) significantly and positively affects safety compliance (C.R. = 3.769). Forming a good safety communication cycle among employees and leaders may enhance employees’ incentives to comply with safety regulations, rules, and operations.

### 3.2. Direct, Indirect, and Overall Effects

Table 4 showed the various effects of LSBs on safety performance. The following conclusions can be drawn:

(1) On all dimensions of LSBs, safety training has the greatest effect on employees’ safety compliance and safety participation.
(2) No dimensions of LSBs had significant effects on safety accidents.
(3) Safety management commitment has no direct, significant, positive impacts on employee safety compliance, safety participation, or safety accidents. Thus, H1(2), H2(2), and H3(2) are not supported. Additionally, safety management commitment has indirect, significant, and positive effects on employees’ safety compliance and safety participation.
(4) Safety incentives have no direct, significant, positive effects on employees’ safety compliance, safety participation, or safety accidents, which rejects H1(3), H2(3), and H3(3). However, safety incentives have indirect, significant, and positive effects on employees’ safety compliance and safety participation.
(5) Safety training and safety communication with feedback have direct, significant, and positive effects on employees’ safety compliance. Safety training and safety policy have direct, significant, and positive effects on employees’ safety participation. Thus, H1(1), H1(5), and H2(1) are supported.

### 3.3. Mediator Effects

According to Figure 4 and Table 3 and Table 4, the mediator effect of the model was analysed as follows:

(1) In this study, the hypothesis that safety communication with feedback will mediate the relationships between safety management commitment and safety compliance, safety participation, and safety accident is not confirmed.
(2) Safety training fully mediates the effects of safety management commitment on safety compliance and safety participation. Via safety training (β = 0.380, *p* = 0.000), safety management commitment positively and significantly affects safety compliance (β = 0.504, *p* = 0.000) and safety participation (β = 0.70, *p* = 0.000). Thus, H4(1) and H5(1) are supported, and H6(1) is not supported.
(3) Safety policy fully mediates the effect of safety management commitment on safety participation. Via safety policy (β = 0.392, *p* = 0.000), safety management commitment has a significant and positive effect on safety participation (β = 0.706, *p* = 0.000). Thus, the results accept H5(2) and reject H4(2) and H6(2).
(4) Safety communication with feedback does not mediate the relationships between safety management commitment and safety compliance, safety participation, or safety accidents. Thus, H4(3), H5(3), and H6(3) are not supported.
(5) Safety training fully mediates the effects of safety incentives on safety compliance and safety participation. Via safety training (β = 0.543, *p* = 0.000), safety incentives have significant and positive effects on safety compliance (β = 0.504, *p* = 0.000) and safety participation (β = 0.706, *p* = 0.000). The results accept H7(1) and H8(1) and reject H9(1).
(6) Safety policy fully mediates the effect of safety incentives on safety participation. Via safety policy (β = 0.453, *p* = 0.000), safety incentives have a significant and positive effect on safety participation (β = 0.706, *p* = 0.000). Thus, H8(2) is supported, and H7(2) and H9(2) are not supported.
(7) Safety communication with feedback fully mediates the effects of safety incentives on safety compliance. Via safety communication with feedback (β = 0.414, *p* = 0.005), safety incentives have a significant and positive effect on safety compliance (β = 0.504, *p* = 0.000). Thus, H7(3) is accepted, and H8(3) and H9(3) are not accepted.

### 3.4. General Conclusions

**Figure 5:**
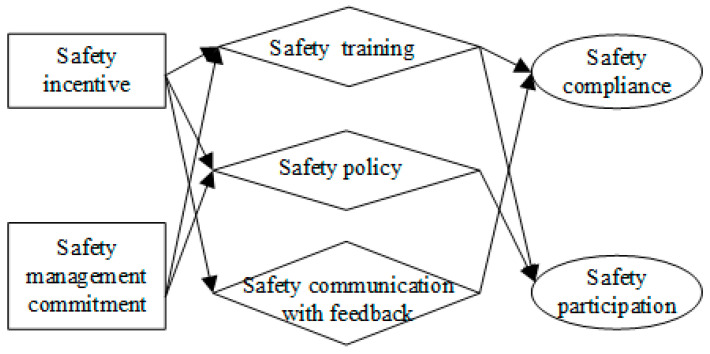
The path of effects on employee safety behaviour.

(1) Leadership safety management commitment, safety incentives, safety training, and safety communication with feedback have significant positive effects on employees’ safety compliance.
(2) Leadership safety management commitment, safety incentives, safety training, and safety policy have significant positive effects on employees’ safety participation.
(3) Leadership safety management commitment, safety incentives, safety training, safety policy, and safety communication with feedback have no significant positive effects on safety accidents.
(4) The overall effects of each variable on safety compliance in descending order are: safety training (0.504 *), safety incentives (0.480 *), safety communication with feedback (0.377 *), safety management commitment (0.281 *), and safety policy (0.110).
(5) The overall effects of each variable on safety participation in descending order are: safety training (0.706 *), safety incentives (0.496 *), safety management commitment (0.365 *), and safety policy (0.247 *).
(6) The overall effects of each variable on safety accidents in descending order are: safety management commitment (0.131), safety communication with feedback (0.111), safety incentives (0.064), and safety policy (0.040).
(7) The path of effects on employees’ safety behaviour is shown in Figure 5.

## 4. Discussion

This study analysed the overall effects of the five dimensions of LSBs on safety performance. The results show that leaders’ safety attitudes and safety behaviours directly affect employees’ safety behaviours and play an important role in safety performance. Meanwhile, the results indicate that safety training, safety policy, and safety communication with feedback are significant mediators in predicting safety performance.

### 4.1. Implications of This Study’s Findings

According to the results, the affecting factors included leaders’ safety training, safety management commitment, safety incentives, safety policy, and safety communication with feedback, which predicted safety performance significantly and positively. However, considering the comprehensive effects on safety performance, safety management commitment and safety incentives were mediated by safety training, safety policy, and safety communication with feedback. According to the affecting path and overall effects, the conclusions are summarized as follows.

#### 4.1.1. The Factors That Affect Employees’ Safety Compliance

In keeping with the findings by Leung et al. [42], leaders’ safety training and safety communication with feedback have direct, significant, and positive effects on employees’ safety compliance. Effective safety training can tell employees what regulations and rules they should comply with, and good communication helps employees increase safety awareness and avoid unsafe behaviours. If leaders provide a variety of safety training opportunities and encourage employees to participate, employees’ safety compliance will be promoted. Leaders could establish smooth communication regarding risks and clear safety goals, which will also effectively promote employees’ safety compliance [16,29,57,58].

Safety management commitment has an indirect, significant, and positive effect on employees’ safety compliance by the mediator of safety training. Positive and convincing safety management commitment can increase employees’ initiative to join safety training and safety activities. Through regular safety training, employees can understand safety regulations and rules more comprehensively and upgrade their skills to avoid unsafe operations, which ultimately strengthens their safety compliance. In addition, when employees behave dangerously, leaders should take measures timely, and enhancing safety training is one of the most important measures in safety management [59]. When accidents happen, leaders need to stress hazard identification skills, which mostly come from safety training [60]. Overall, leaders’ safety management commitments, such as attitudes towards complying with safety rules and regulations, and dealing with safety issues, are embodied in safety trainings, which ultimately affect employees’ safety compliance.

Safety incentives have an indirect, significant, and positive effect on employees’ safety compliance mediated by safety training and safety communication with feedback, which is different from Lu and Yang [15]. The difference may be associated with the conditions of mining in China. Although leaders reward employees who do well in safety behaviour, the evaluation standard is zero accidents and the realization of production targets. This stance does not reward or encourage employees’ safe work; in contrast, it may promote them to hide hazards in order to obtain more rewards [61]. Thus, safety incentives have no direct effect on safety compliance in this study.

Secondly, according to incentive theory, expectation theory, and goal-setting theory, with appropriate safety incentives, employees hope to improve their operation skills, safety consciousness, and safety performance by taking part in various trainings. Given this, leaders should conduct effective safety training and good safety communication to meet employees’ safety needs, which can increase employees’ desire to act more safely and their safety compliance. In addition, in the workplace, if leaders timely praise employees for their risk identification, rather than habitually criticizing employees for their mistakes, such positive reinforcement can promote employees’ safety work advantageously. Employees expect to communicate risks and accidents with leaders, and communications about safety aims can motivate employees’ safety performance. Overall, these results confirm incentive theory to a certain degree.

Different from previous studies [15,33,37,50,62], the results showed that safety policy had a positive effect on safety compliance which was not significant. Safety policy includes two aspects, emphasis on site safety and establishment of safety responsibility systems [63]. Leaders always focus on the safety status of underground operations. Although leaders inspect workplaces every day, this inspection temporarily promotes safe work. As for employees, this is a passive promotion because employees’ safety awareness is the root cause for safety work [64]. Secondly, even when leaders establish a sound system of safety responsibility, how to effectively implement safety responsibility is more important. To encourage employees’ responsibility, it is more crucial to reward employees in accordance with safety requirements [17]. This positive reinforcement has more significance for safety compliance. Thus, the effect of safety policy on safety compliance is not significant in our study.

#### 4.1.2. The Factors That Affect Employee Participation

Safety training and safety policy of leadership have direct, significant, and positive effects on employees’ safety participation. Safety training can not only improve employees’ safety awareness, integration in the enterprise, and understanding of safety attitudes but also encourage employees to comply with mutual responsibility systems. The same as previous studies [15,62], for safety policy, leaders should establish safety responsibility systems and make clear safety accountability at all levels, which can further strengthen employees’ responsibility for their own behaviours. The responsibility employees take will increase their attention to daily safety training and increase their initiative to participate in safety activities.

Safety management commitment has an indirect, significant, and positive effect on employees’ safety participation mediated by safety training and safety policy. Safety management commitment has no direct and positive effect on safety participation, which is different from Lu [15]. In the survey of enterprises, even when leaders have clear safety attitudes and timely correct employees’ unsafe behaviours, employees have little chance to participate in safety management. Thus, these activities have little direct impact on employees’ participation in safety activities and meetings or on promoting workplace safety. In contrast, according to the results above, if leaders maintain emphasis on safety, deal with accidents timely, and take the lead in observing safety regulations, their efforts will accentuate the effects of conducting safety training and safety policy. Finally, employees’ safety participation will improve.

Safety incentives have indirect, significant, and positive effects on employees’ safety participation mediated by safety training and safety policy. Although leaders reward those who set examples of safety, the rewards are based on indicators of accidents rather than on employees’ participation in safety activities. As such, safety incentives affect safety participation indirectly. Proper and adequate safety incentives for employees can determine the effects of safety management activities, such as carrying out safety training and enacting safety policy. These activities can enhance employees’ safety awareness and encourage employees to actively participate in safety management [65]. In emphasizing site safety, it is also important to encourage employees to ensure the safety of colleagues.

Previous studies showed that effective safety communication with feedback can significantly promote safety participation [16,57]. However, safety communication with feedback has a positive but nonsignificant effect on employees’ safety participation. The causes of this phenomenon are mainly features inherent to the mining industry. Miners, managers, and leaders occasionally communicate with each other, so the influence on safety participation exists, but it is not significant, which may be different from other studies.

#### 4.1.3. The Factors That Affect Safety Accidents

Safety management commitment, safety policy, and safety communication with feedback have positive effects on safety accidents (fewer accidents are indicated by higher scores), but not significantly. The influence from safety management commitment is the greatest. This result is consistent with previous research [3,32,33,34,66,67]. Safety management commitment is perceived by employees as leaders’ attitudes and methods of dealing with safety problems. Leaders’ positive behaviours, such as handling risks seriously and timely, can possibly reduce accidents. In safety policy, if leaders emphasize practical workplace safety and set up effective safety responsibility systems, the efforts will decrease accidents [68]. Safety communication with feedback is a beforehand control measure perceived by employees [69]. Whether employees communicate hazards and risk information before accidents, whether leaders encourage employees to report dangers, and whether leaders propose clear safety goals and requirements can all predict safety accidents. Finally, different from previous research, safety incentives have no influence on safety accidents. The main reason is that valid safety incentives are lacking in mining enterprises.

### 4.2. Limitations and Future Research

Even after the exploratory factor analysis, the LSB questionnaire items mainly referred to existing scales, but there is a lack of research on whether these items conform to the situation of the mining industry in China. Therefore, it is hoped that future surveys will better reflect the actual circumstances of mining in China.

Owing to the time and cost, the study only surveyed a few representative mining enterprises. Although the conclusions are more targeted, the results have limitations in overall representativeness. In future studies, we can survey more enterprises.

To date, the studies on LSBs and employees’ safety behaviours have almost all adopted self-report measures, and this study is no exception. As a result, the findings are very dependent on the integrity of the respondents, and some respondents may answer the questions casually and unreasonably. Therefore, in order to understand the behaviours of mining enterprises and their affecting factors more accurately, other methods should be used such as interviews and behavioural experiments.

## 5. Conclusions

Regression analysis and structural equation modelling analysis were used to examine the cause-and-effect relationships between leadership safety behaviours and safety performance. Data collected from a survey of 305 miners were analysed by exploratory factor analysis and confirmatory factor analysis. The following conclusions may be drawn from the results:

(1) Safety management commitment, safety incentives, safety training, and safety communication with feedback have significant positive effects on employees’ safety compliance.
(2) Safety management commitment, safety incentives, safety training, and safety policy have significant positive effects on employees’ safety participation.
(3) The overall effects of each variable on safety compliance in descending order are: safety training (0.504 *), safety incentives (0.480 *), safety communication with feedback (0.377 *), safety management commitment (0.281 *), and safety policy (0.110).
(4) The overall effects of each variable on safety participation in descending order are: safety training (0.706 *), safety incentives (0.496 *), safety management commitment (0.365 *), and safety policy (0.247 *).
(5) The overall effects of each variable on safety accidents in descending order are: safety management commitment (0.131), safety communication with feedback (0.111), safety incentives (0.064), and safety policy (0.040).
(6) Safety management commitment and safety incentives predicted employees’ improved safety behaviours, but this influence was mediated by safety training, safety policy, and safety communication with feedback.

In conclusion, we established a partial mediation model of leadership behaviours’ effects on safety performance, and we analysed the effects of leadership behaviours’ five dimensions on safety performance’s three dimensions. The results provide greater insights into the value of increasing safety performance in mining enterprises. Understanding how leadership behaviours affect employees’ safety performance will help leaders to effectively improve safety management behaviours.

## Figures and Tables

**Table 1:** The simple linear regression analysis coefficients of the five dimensions of LSBs.

Model	Independent Variable	*R*	*R* ^2^	Δ*F*	*F*	Beta	*t*	Sig.
1	ST	0.875	0.765	455.192 ***	455.192 ***	0.875	21.335 ***	0.000
1	SMC	0.790	0.625	233.100 ***	233.100 ***	0.790	15.268 ***	0.000
1	SI	0.704	0.496	137.810 ***	137.810 ***	0.704	11.739 ***	0.000
1	SP	0.706	0.499	139.318 ***	139.318 ***	0.706	11.803 ***	0.000
1	SCF	0.501	0.251	46.801 ***	46.801 ***	0.501	6.841 ***	0.000

*** At the 0.001 level (three tailed), the correlation is significant.

**Table 2:** The stepwise multiple regression analysis coefficients of the five dimensions of LSBs.

Input Variable Order	*R*	*R* ^2^	*F*	Δ*F*	*B*	Beta	TOL	VIF	Eigen-Values	CI
ST	0.723	0.523	153.590 ***	153.590 ***	0.800	0.554	0.625	1.599	0.071	7.422
SCF	0.754	0.569	91.749 ***	14.785 ***	0.599	0.231	0.862	1.160	0.017	15.155
SP	0.765	0.585	64.879 ***	5.370 *	0.575	0.152	0.695	1.439	0.013	17.491

*** At the 0.001 level (three tailed), the correlation is significant. * At the 0.05 level (three tailed), the correlation is significant.

**Table 3:** The standardized path coefficients of the second model.

Internal Latent Variable		Exogenous Latent Variable	Regression Weights	C.R.	*p*	Standardized Regression Weights
SP	<---	SMC	0.438	4.001	***	0.392
SCF	<---	SMC	0.140	1.066	0.286	0.124
ST	<---	SI	0.567	5.456	***	0.543
SP	<---	SI	0.441	4.488	***	0.453
SCF	<---	SI	0.408	2.821	0.005	0.414
ST	<---	SMC	0.455	4.314	***	0.380
Safety compliance	<---	ST	0.630	4.952	***	0.504
Safety participation	<---	ST	0.607	6.575	***	0.706
Safety compliance	<---	SP	0.147	1.265	0.206	0.110
Safety participation	<---	SP	0.227	3.051	0.002	0.247
Safety accident	<---	SP	0.046	0.364	0.715	0.040
Safety compliance	<---	SCF	0.499	3.769	***	0.377
Safety accident	<---	SMC	0.129	0.950	0.342	0.101
Safety accident	<---	SCF	0.125	1.210	0.226	0.111

*** At the 0.001 level (two tailed), the correlation is significant.

**Table 4:** The direct, indirect, and overall effects of LSBs on safety performance.

Variable	Safety Compliance			Safety Participation			Safety Accident		
	Direct Effect	Indirect Effect	Overall Effect	Direct Effect	Indirect Effect	Overall Effect	Direct Effect	Indirect Effect	Overall Effect
Mediator variable									
ST	0.504 *	—	0.504 *	0.706 *	—	0.706 *	—	—	—
SP	0.110	—	0.110	0.247 *	—	0.247 *	0.040	—	0.040
SCF	0.377 *	—	0.377 *	—	—	—	0.111	—	0.111
Exogenous variable									
SMC	—	0.281 *	0.281 *	—	0.365 *	0.365 *	0.101	0.029	0.131
SI	—	0.480 *	0.480 *	—	0.496 *	0.496 *	—	0.064	0.064

Note: “—” means no effect. * At the 0.05 level (one tailed), the correlation is significant.

## Data Availability

Not applicable.

## Appendix A

**Table A1:** Items on the leadership safety behaviours questionnaire.

**Management commitment**	
1	Leadership attaches great importance to safety issues
2	Safety rules and procedures are strictly followed by leaders
3	Corrective action is always taken when the leaders are told about unsafe practices
4	In my workplace, managers/supervisors do not show interest in the safety of workers
5	Leaders consider safety to be equally important with production
6	Members of leadership do not attend safety meetings
7	I feel that leaders are willing to compromise on safety to increase production
8	When near-miss accidents are reported, my leaders act quickly to solve the problems
9	My leaders provide sufficient personal protective equipment for the workers
**Safety communication with feedback**	
1	My company doesn’t have a hazard reporting system where employees can communicate hazard information before incidents occur
2	Leaders operate an open-door policy on safety issues
3	There is sufficient opportunity to discuss and deal with safety issues in meetings
4	The target and goals for safety performance in my organization are not clear to the workers
5	There are open communications about safety issues in this workplace
**Safety policy**	
1	My leaders explain the safety policy clearly
2	My leaders emphasize worksite safety
3	My leaders have established a safety responsibility system
4	My leaders establish clear safety goals
**Safety incentive**	
1	My leaders reward those who set an example in safety behaviour
2	My leaders praise workers’ safety behaviour
3	My leaders have set up a safety incentive system
4	My leaders encourage workers to report potential incidents without punishment
5	My leaders encourage workers to provide safety suggestions
6	My leaders trust workers
**Safety training**	
1	My company gives comprehensive training about health and safety issues to the employees in the workplace
2	New recruits are trained adequately in safety rules and procedures
3	Safety issues are given high priority in training programs
4	I am not adequately trained to respond to emergency situations in my workplace
5	Leaders encourages workers to attend safety training programs
6	Safety training given to me is adequate to enable to me to assess hazards in workplace

## Appendix B

**Table A2:** Items of safety performance questionnaire.

**Safety compliance**	
1	I maintain safety awareness at work
2	I do not neglect safety, even when in a rush
3	I comply with safety rules and standard operational procedures
4	I wear personal protective equipment at work
5	In order to complete more work to get more piece-rate income or measurement of income, I may ignore safety
**Safety participation**	
1	I actively participate in safety meetings
2	I encourage my co-workers to work safely
3	I voluntarily carry out tasks or activities that help to improve workplace safety
**Safety accidents**	
1	In the past three years, I had an accident
